# Physiological Mechanisms Underlying Maize Yield Enhancement by Straw Return in the Thin-Layer Mollisol Region of the Songnen Plain

**DOI:** 10.3390/plants14213331

**Published:** 2025-10-31

**Authors:** Chenglong Guan, Tai Ma, Ming Miao, Jiuhui Chen, Zhicheng Bao, Baoyu Chen, Jingkun Lu, Fangming Liu, Nan Wang, Hongjun Wang, Zhian Zhang

**Affiliations:** 1 Agronomy College, Jilin Agricultural University, Changchun 130118, China; 15144441522@163.com (C.G.); m3832771284@163.com (T.M.); 15698259707@163.com (J.C.); 20241837@mails.jlau.edu.cn (Z.B.); 2 Jilin Academy of Agricultural Sciences (Northeast Agricultural Research Center of China), Changchun 130033, China; bych76@jaas.com.cn (B.C.); lujingkun5258@163.com (J.L.); liufangming924@126.com (F.L.); wangnan0307@163.com (N.W.); 3 Jilin Academy of Agricultural Sciences, Jilin 132011, China; miaoming2000@163.com

**Keywords:** straw return method, root bleeding sap, photosynthetic characteristics, matter accumulation, maize

## Abstract

Long-term intensive cultivation has caused soil fertility decline and structural degradation in the Songnen Plain, thereby constraining maize root development and yield formation. As a fundamental conservation tillage practice, straw return enhances soil function by incorporating exogenous organic matter and regulating root-shoot physiological processes. However, the mechanism underlying yield improvement through root–photosynthesis–nitrogen synergy remains insufficiently understood. A field experiment was conducted to assess the effects of conventional tillage (CT), straw incorporation (SI), straw mulching (SM), and deep straw incorporation (DF) on maize physiological traits and yield. Compared with CT, DF markedly enhanced root morphology and physiology, increasing the root length, surface area, volume, and root-shoot ratio by 16.46%, 23.87%, 26.64%, and 51.34%, respectively. The root bleeding intensity increased by 23.63%, whereas amino acid and nitrate contents in the bleeding sap increased by 29.20% and 65.93%, respectively, indicating improved root nutrient transport capacity. The enhanced root system positively influenced shoot photosynthesis by increasing the chlorophyll SPAD value by 16.05%, net photosynthetic rate (P_n_) by 11.28%, and the activities of RuBP, PEP, nitrate reductase (NR), and glutamine synthetase (GS) by 10.59%, 24.36%, 29.94%, and 12.47%, respectively. These synergistic improvements significantly promoted post-anthesis biomass accumulation and yield formation. DF increased nitrogen and dry matter accumulation at the R3 stage by 26.61% and 15.67%, respectively, and resulted in an average yield increase of 8.34%, which was primarily due to an 11.96% increase in 100-grain weight. Although SI and SM also improved certain physiological indices, their effects were weaker than those of DF. RF analysis identified sap nitrate content (RNO), bleeding intensity (RBI), root length (RL), and root volume (RV) as key yield determinants. PLS-SEM further revealed that straw return enhanced root morphology and bleeding traits (path coefficients: 0.96 and 0.82), which subsequently improved leaf photosynthetic traits (path coefficients: 0.52 and 0.39) and biomass accumulation (path coefficient: 0.71). Collectively, these improvements promoted post-anthesis nitrogen accumulation and dry matter partitioning into grains. These findings elucidated the physiological mechanism by which deep straw incorporation increased maize yield through root system optimization, providing a theoretical basis for conservation tillage optimization in the thin-layer Mollisol region of the Songnen Plain.

## 1. Introduction

Maize (*Zea mays* L.) is a staple crop worldwide, and its production stability is fundamental to global food security [1]. In the thin-layer Mollisol region of the Songnen Plain, Northeast China, intensive cultivation over decades has led to the plow layer thinning, nutrient depletion, and soil structural deterioration, which together restrict sustainable maize productivity [2]. As a central measure in conservation tillage, straw return can improve soil structure, enhance yield, and increase water and fertilizer efficiency through resource recycling [3,4]. Most previous studies have focused on soil physicochemical properties or aboveground growth responses [5,6]. However, systematic investigations of the synergistic mechanisms connecting root function (such as bleeding) with canopy photosynthesis under different straw return strategies remain limited.

Root bleeding traits, which have been widely studied since the 1990s in plant physiology and hormone transport, reflect the intensity of root physiological activity. However, their specific contribution to the straw return-root-photosynthesis synergy is still unclear in thin-layer Mollisols. Roots are the primary organs for nutrient and water acquisition. Bleeding intensity and composition reveal physiological activity, whereas morphological features determine the spatial efficiency of resource capture [7,8]. Nitrogen serves as a critical nutrient and drives growth, while its accumulation and redistribution among vegetative organs and grains directly affect yield components [9]. Increasing evidence indicates that root bleeding intensity and morphology are tightly linked to photosynthetic performance, influencing carbon assimilation and nitrogen accumulation through enhanced chlorophyll content, higher photosynthetic rate, and greater carbon-nitrogen enzyme activity [10,11]. Recent studies have reported substantial effects of straw return on root morphology in major crops. For instance, straw mulching expands root surface area in wheat, whereas deep incorporation promotes deeper root penetration in rice [5,12]. However, the responses of root physiological functions, with bleeding traits serving as critical indicators of root activity and nutrient transport, remain insufficiently elucidated under different straw return methods [13,14]. Straw return may enhance bleeding intensity, root morphological development, and nitrogen acquisition efficiency by improving the rhizosphere environment, thereby elevating canopy photosynthetic traits [15]. Nevertheless, most existing findings have been limited to isolated indices, lacking comprehensive analyses of bleeding characteristics, morphology, photosynthesis, nitrogen dynamics, and yield under multiple return methods in the Songnen Plain [16]. Such an integrated evaluation is essential to reveal the physiological mechanisms by which straw return regulates root-canopy interactions to improve yield and resource-use efficiency [12,17]. Moreover, the relationships between bleeding traits, root morphology, and nitrogen translocation remain largely qualitative, with limited multi-year datasets. The differences among return methods in regulating these processes also require further exploration, and multi-season experiments are needed to determine optimal practices for yield improvement and efficient resource utilization [18,19]. These gaps hinder the complete understanding of how straw return enhances maize productivity through mechanisms such as root bleeding.

This study aimed to systematically clarify the physiological mechanisms by which different straw return methods regulate root architecture and bleeding traits and influence canopy carbon assimilation, nitrogen metabolism, and yield formation. A continuous response pathway was established from root morphogenesis and physiological function to leaf photosynthesis and enzyme activity, and further to nitrogen uptake and dry matter accumulation, thereby constructing a framework for straw management–root/shoot interaction–yield formation. This study aimed to investigate the short-term effects of various straw incorporation practices on root growth, photosynthetic performance, and nitrogen accumulation, with a two-year experimental period considered appropriate for evaluating these initial responses. We conducted a two-year field experiment in Gongzhuling, Jilin Province (2023–2024), involving four treatments: conventional tillage (CT), straw incorporation (SI), straw mulching (SM), and deep straw incorporation (DF). The objectives were to (1) evaluate the effects of different straw return methods on root bleeding traits, root morphology, and canopy photosynthetic performance and to identify their relationships with key carbon and nitrogen metabolic enzymes; (2) determine the contributions of root bleeding and morphological traits to nitrogen uptake, dry matter accumulation, and yield formation; and (3) elucidate the pathways through which straw return could affect yield components via carbon assimilation, nitrogen metabolism, and biomass allocation. This study provides mechanistic evidence that optimized root traits under straw return, particularly DF, enhance canopy photosynthetic efficiency (e.g., chlorophyll content, photosynthetic rate, and RuBP and PEP activity) and nitrogen metabolism (e.g., nitrate reductase and glutamine synthetase activity), thereby promoting nitrogen accumulation and dry matter partitioning after anthesis to increase yield. The results could establish a theoretical foundation for efficient straw return technologies to improve maize yield and resource-use efficiency in the thin-layer Mollisol region of Northeast China.

## 2. Results

### 2.1. Effects of Different Straw Return Methods on Root Morphology and Physiological Traits

**Figure 1:**
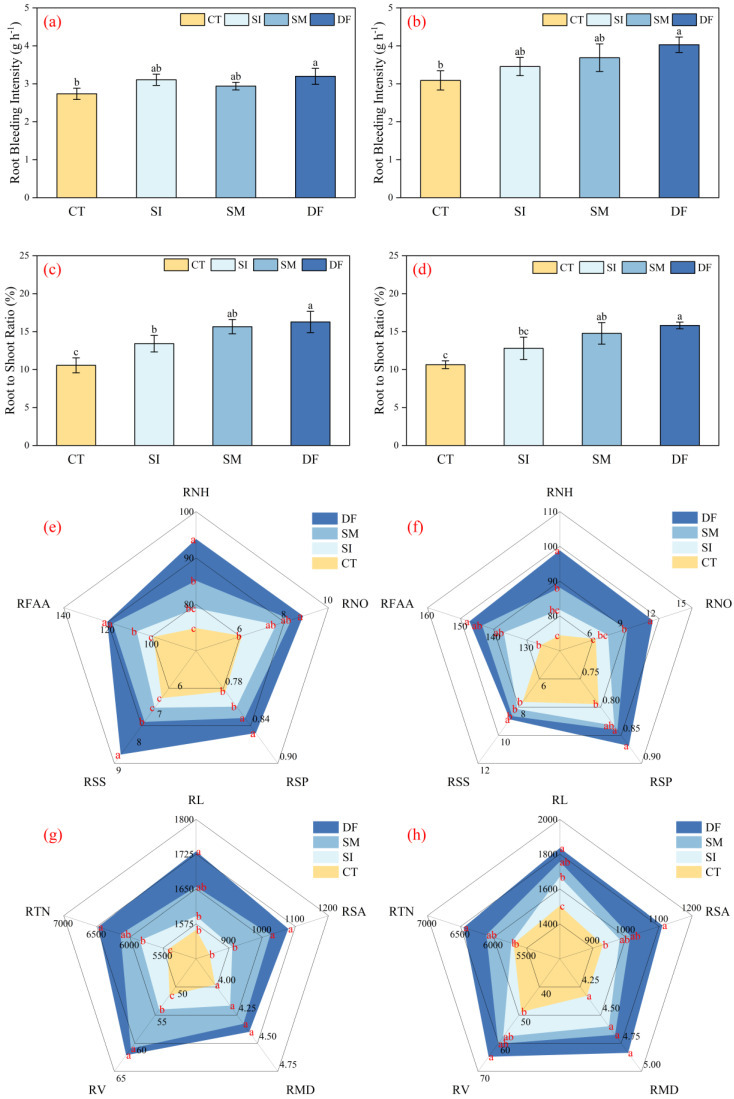
Root bleeding intensity in 2023 and 2024 (**a**,**b**), root-shoot ratio in 2023 and 2024 (**c**,**d**), bleeding sap components in 2023 and 2024 (**e**,**f**), and root morphology in 2023 and 2024 (**g**,**h**). Different lowercase letters indicate significant differences (*p* < 0.05), the same applies below. Abbreviations: RNH (NH_4_^+^ μg·mL^−1^), RNO (NO_3_^−^ μg·mL^−1^), RSP (soluble protein mg·mL^−1^), RSS (soluble sugar mg·mL^−1^), RFAA (free amino acids μg·mL^−1^); root morphology: RL (length cm), RSA (surface area cm^2^), RMD (mean diameter mm), RV (volume cm^3^), RTN (tip number).

Straw return treatments exerted significant influences on maize root physiology and morphology (Figure 1). For physiological function (Figure 1a,b), DF markedly increased root bleeding intensity by 16.89% and 30.42% compared with CT over two years (*p* < 0.05). The root–shoot ratio also consistently increased (Figure 1c,d). In 2023, SI, SM, and DF increased it by 27.06%, 48.21%, and 54.10%, respectively, relative to CT. In 2024, SM and DF raised it by 38.79% and 48.58% (*p* < 0.05).

Analysis of bleeding sap composition revealed clear and significant differences (*p* < 0.05) among the treatments (Figure 1e,f). Across the two-year experiment, the RNH content under DF and SM treatments was significantly (*p* < 0.05) higher than that under CT, with average increases of 29.19% and 16.00%, respectively. No significant difference was observed between SI and CT. For RNO content, DF exhibited a significant (*p* < 0.05) increase of 45.87% over CT in 2023, whereas SM and SI showed no significant differences compared with CT. In 2024, both DF and SM significantly (*p* < 0.05) increased RNO content relative to CT by 86.00% and 47.30%, respectively, whereas SI remained statistically similar to CT. Regarding RSP content, both DF and SM maintained significantly (*p* < 0.05) higher levels than CT in both years, with average increases of 8.90% and 5.60%, respectively; again, SI did not differ significantly from CT. In terms of RSS content in 2023, DF was significantly (*p* < 0.05) higher than that of SM, SI, and CT, with increases of 15.44%, 23.69%, and 30.06%, respectively, whereas SI and CT did not differ significantly. In 2024, DF remained significantly (*p* < 0.05) higher than SM, SI, and CT, with increases of 2.68%, 9.06%, and 16.42%, respectively; however, SM, SI, and CT showed no significant differences among them. For RFAA content in 2023, DF was significantly (*p* < 0.05) higher than SI and CT by 12.51% and 21.08%, respectively, but did not differ significantly from that of SM. In 2024, DF remained significantly (*p* < 0.05) higher than CT by 17.30%, whereas no significant differences were observed among DF, SM, and SI.

With respect to root morphology (Figure 1g,h), DF strongly promoted root development. In 2023, the indices of root length, surface area, volume, and tip number increased by 10.80%, 28.39%, 20.64%, and 19.37%, respectively, compared with CT (*p* < 0.05). In 2024, these increments were 22.12%, 19.36%, 32.64%, and 13.27%, respectively (*p* < 0.05). SM enhanced the root surface area and volume but less effectively than DF, while no significant differences in root diameter were observed among treatments in either year.

These results demonstrated that DF optimized maize root development by improving root architecture, enhancing physiological activity, and strengthening nutrient transport, thereby establishing a foundation for enhanced canopy growth and yield formation.

### 2.2. Leaf Photosynthetic Characteristics and Carbon–Nitrogen Metabolic Enzyme Responses to Straw Return Methods

**Figure 2:**
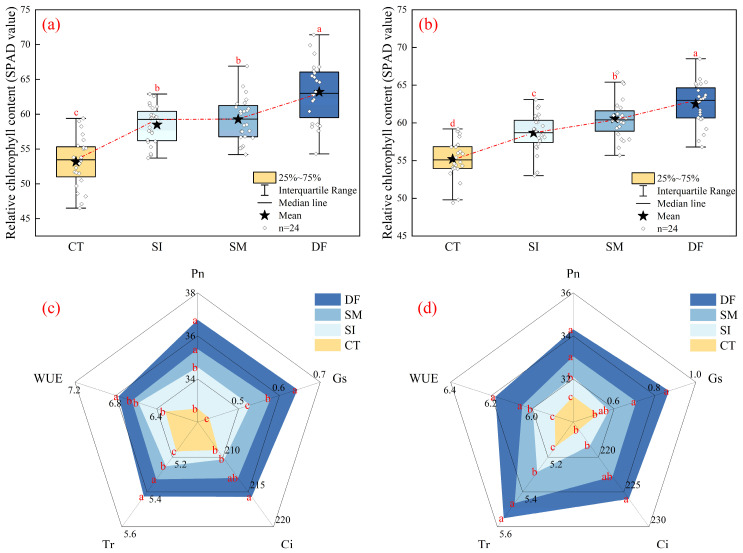
Chlorophyll Relative Content and Photosynthetic Characteristics. Relative chlorophyll content (SPAD) in 2023 and 2024 (**a**,**b**) and photosynthetic parameters in 2023 and 2024 (**c**,**d**). Abbreviations: Pn, net photosynthetic rate (μmol·m^−2^·s^−1^); Gs, stomatal conductance (mol·m^−2^·s^−1^); Ci, intercellular CO_2_ concentration (μmol·mol^−1^); Tr, transpiration rate (mmol·m^−2^·s^−1^); WUE, leaf water use efficiency (μmol·mmol^−1^).

Straw return treatments significantly affected the photosynthetic performance of maize leaves (Figure 2). In the 2023 trial, the SPAD value under the DF treatment was significantly (*p* < 0.05) higher than those under SM, SI, and CT, with average increases of 6.65%, 8.03%, and 18.86%, respectively. Meanwhile, both SM and SI treatments also showed significantly (*p* < 0.05) higher SPAD values than CT, with average increases of 11.44% and 10.02%, respectively. In the 2024 trial, the SPAD value under DF treatment remained significantly (*p* < 0.05) higher than those under SM, SI, and CT, with average increases of 3.20%, 6.52%, and 13.25%, respectively. In addition, the SPAD value under SM treatment was significantly (*p* < 0.05) higher than that under SI, with an average increase of 3.21%.

DF substantially enhanced carbon assimilation capacity (Figure 2c,d). Net photosynthetic rate (P_n_) increased by 12.62% and 9.94% over CT during the two years, while SM produced moderate improvements. Stomatal conductance (G_s_) increased by 50.38% and 67.03% under DF and by 35.12% and 36.18% under SM. Intercellular CO_2_ concentration (C_i_) and transpiration rate (T_r_) also increased under DF, with Ci increasing by 3.20% and 4.56% and Tr by 4.99% and 7.94% (*p* < 0.05). SM displayed similar but weaker effects. Water use efficiency (WUE) increased significantly under DF by 7.27% and 4.93% (*p* < 0.05), with SM also providing improvement.

Overall, all straw return methods improved photosynthetic traits, but DF demonstrated the most stable and pronounced effects, followed by SM, while SI remained the least effective. By enhancing chlorophyll content, stomatal conductance, carbon assimilation efficiency, and water use efficiency, particularly under DF, straw return collectively advanced photosynthetic performance.

Different straw return modes significantly enhanced the activities of key enzymes involved in carbon and nitrogen metabolism in maize leaves (Table 1). For carbon metabolism enzymes, the activity of RuBP carboxylase (RuBP) under SM and DF treatments increased by 17.59% and 9.02% compared with CT in 2023 and by 27.53% and 12.17% in 2024 (*p* < 0.05). PEP carboxylase (PEP) activity under SL, SM, and DF treatments increased by 18.06%, 11.13%, and 33.21% relative to CT in 2023 and by 10.57%, 9.48%, and 15.52% in 2024 (*p* < 0.05). For nitrogen metabolism enzymes, nitrate reductase (NR) activity under SL, SM, and DF treatments increased significantly by 23.23%, 16.37%, and 34.52% compared with CT in 2023 and by 14.85%, 14.85%, and 25.37% in 2024 (*p* < 0.05). Glutamine synthetase (GS) activity under SM and DF treatments increased by 6.89% and 9.11% compared with CT in 2023, whereas in 2024, the increases under SL, SM, and DF were 5.63%, 8.63%, and 15.84%, respectively (*p* < 0.05). The year × treatment interaction had no significant effect on RuBP, NR, or GS activity (*p* > 0.05), indicating that the treatment effects were stable across years.

### 2.3. Plant Nitrogen Accumulation and Translocation in Response to Straw Return

**Figure 3:**
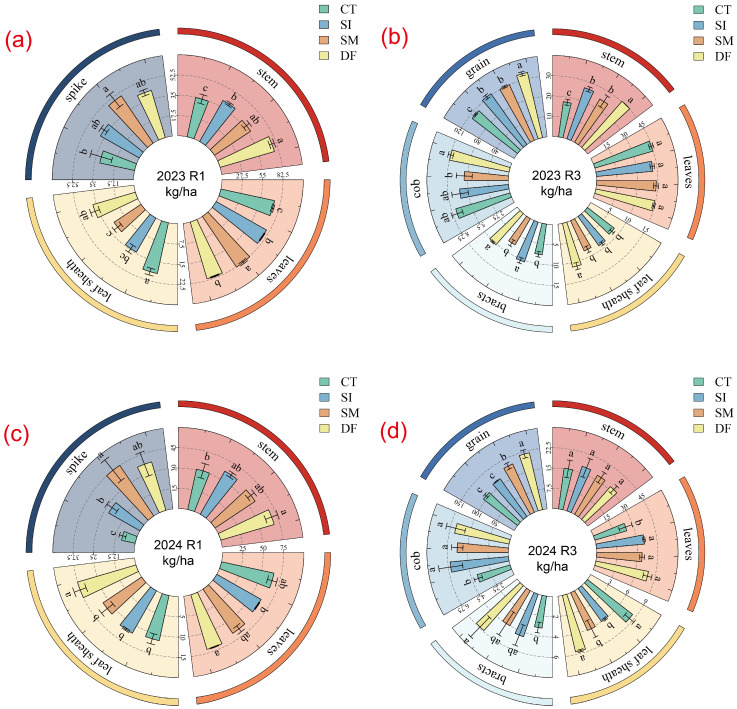
Effects of straw return methods on nitrogen accumulation in maize plants. Nitrogen accumulation at the R1 and R3 stages in 2023 (**a**,**b**) and nitrogen accumulation at the R1 and R3 stages in 2024 (**c**,**d**). Plant organs include stems, leaves, leaf sheaths, spikes, bracts, cobs, and grains.

Different straw return methods increased nitrogen accumulation in maize plants (Figure 3), mainly in the leaves at the R1 stage, followed by the stem, spikes, and leaf sheaths. At the R1 stage in 2023 (Figure 3a), DF, SM, and SI treatments significantly increased the leaf nitrogen content by 8.24%, 11.89%, and 5.55%, respectively, compared with CT. By the R3 stage, nitrogen accumulation had shifted predominantly toward the grains. In contrast, at the R1 stage in 2023 (Figure 3b), the leaf nitrogen content under DF, SM, and SI treatments remained significantly higher than that under CT, with increases of 16.96%, 6.78%, and 8.81%, respectively. Similarly, at the R1 stage in 2024 (Figure 3c), plant nitrogen was still mainly concentrated in the leaves, where the DF treatment led to a significant 6.56% increase in leaf nitrogen content compared with SI. In contrast, at the R3 stage in 2024 (Figure 3d), nitrogen was translocated to the grains. At this stage, the DF treatment resulted in significantly higher nitrogen accumulation than SM, SI, and CT by 10.83%, 27.66%, and 36.05%, respectively. Moreover, the SM treatment also performed significantly better than SI and CT, with increases of 14.40% and 23.60%, respectively.

Biomass nitrogen accumulation also increased under straw return treatments (Table 2). The pre-anthesis accumulation ranked DF > SM ≈ SI > CT, with DF indicating increments of 13.41% and 17.07% over CT in 2023 and 2024, respectively. Post-anthesis accumulation was the greatest under DF (21.70% and 34.76% over CT), with SM also producing notable increases. However, the amount and efficiency of nitrogen translocation did not differ significantly among treatments. It was found that straw return increased total nitrogen storage without altering transformation efficiency, as evidenced by the non-significant year × treatment interaction (*p* > 0.05) for both nitrogen translocation amount and translocation rate.

### 2.4. Dry Matter Accumulation and Yield Response to Straw Return

**Figure 4:**
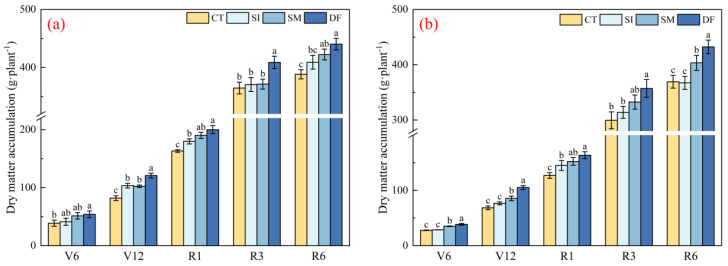
Dry matter accumulation. (**a**,**b**) Dry matter accumulation in 2023 and 2024, respectively.

Straw return markedly promoted dry matter accumulation and yield formation (*p* < 0.05). DF most consistently enhanced the dry matter accumulation at all growth stages (Figure 4a). During vegetative growth (V6–V12), DF increased accumulation by 39.06% and 47.39% in 2023 and by 38.74% and 55.54% in 2024 compared with CT. During reproductive growth (R1–R6), DF also maintained its highest accumulation, increasing by 22.56% and 28.89% at R1 and by 13.37% and 17.07% at R6 across the two years. SM also improved accumulation, although it was less effective than DF. These results indicated that straw return, particularly DF, enhanced dry matter accumulation throughout the growth cycle, which could provide a basis for yield improvement.

Grain yield was significantly increased by straw return, with DF consistently presenting the strongest effect (Table 3). DF increased yield by 4.83% and 11.85% over CT across the two years, whereas SM also improved yield in 2024 (6.22% increase). SI produced only minor effects. The year × treatment interactions were significant (*p* < 0.05), indicating that the effects of the treatments on crop yield varied significantly between years. Yield component analysis indicated that the increases were mainly attributable to greater 100-grain weight. In 2023, SI, SM, and DF increased the 100-kernel weight by 6.07%, 8.28%, and 9.63%, respectively. In 2024, DF increased it by 14.30%. A significant year × treatment interaction was observed for 100-grain weight (*p* < 0.05), and the change depended on both the year and treatment.

### 2.5. Drivers and Regulatory Pathways of Yield Formation

**Figure 5:**
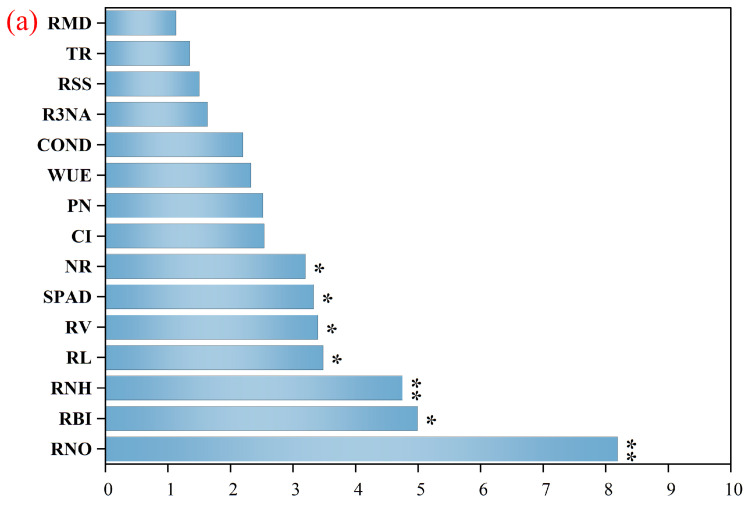

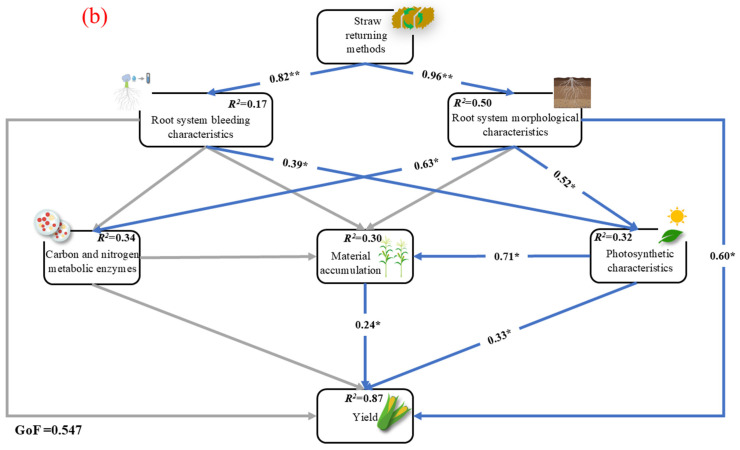
Random Forest and PLS-SEM. (**a**) Random Forest analysis of yield drivers under different treatments. (**b**) Partial least squares path model (PLS-PM) illustrating the relationships among root bleeding traits, root morphological characteristics, carbon–nitrogen metabolic enzyme activities, biomass accumulation, photosynthetic performance, and yield across the four tillage practices. Blue arrows denote significant positive paths. Numbers adjacent to the arrows represent path coefficients. Statistical significance is indicated by * *p* < 0.05 and ** *p* < 0.01. Model goodness-of-fit (GoF) = 0.547.

Random forest (RF) analysis identified root nitrate transport capacity (RNO), bleeding intensity (RBI), sap amino acid content (RNH), root length (RL), chlorophyll content (SPAD), and root volume (RV) as the principal yield-driving factors (Figure 5a). Structural equation modeling (SEM) yielded a goodness-of-fit value of 0.547. SEM analysis indicated that straw return enhanced yield primarily by improving bleeding traits and root morphology (Figure 5b). These root improvements subsequently enhanced the photosynthetic performance, which directly and indirectly (via biomass accumulation) contributed to yield formation. The model revealed a cascade pathway of “root function–canopy photosynthesis–biomass production–yield formation” under straw return practices.

## 3. Discussion

### 3.1. Effects of Straw Return on the Root System: Synergistic Optimization of Morphology and Physiology

As a sustainable agricultural practice, straw return can improve soil quality through multiple pathways, thereby regulating crop root growth and physiological performance [20]. Root bleeding sap exuded from cut roots serves as a key indicator of root water absorption and physiological activity [21,22]. Appropriate straw return practices promote root biomass accumulation and elongation [23]. Previous research has demonstrated that straw return can alter root morphology by increasing diameter, enlarging xylem vessels, and reducing cortical thickness, thereby improving water and nutrient acquisition efficiency in wheat [5]. Studies have shown that enhancing soil physicochemical properties, particularly physical attributes such as porosity and moisture content, through various straw incorporation methods can greatly stimulate the growth and development of plant root systems underground [24]. In this study, different return methods, particularly DF, significantly enhanced the bleeding intensity, root-shoot ratio, and concentrations of nitrogen forms (ammonium and nitrate nitrogen), soluble protein, sugar, and free amino acids in the bleeding sap. These changes indicated that straw return not only improved biomass allocation but also substantially strengthened root physiological activity and nutrient transport. Concurrently, root morphological indices, including total length, surface area, and volume, were markedly increased under straw return. The underlying mechanism may be that enhanced root morphology expands the capacity for water and nutrient acquisition, which further increases bleeding intensity [25]. Moreover, the elevated concentrations of amino acids and nutrients in the bleeding sap reflect enhanced vitality and metabolic activity [26]. Sun et al. [27] reported that higher bleeding intensity in rice promoted the dry matter and nitrogen accumulation, which improved grain yield. Wen et al. [28] observed that maize adjusted its root morphology in response to shoot nutrient supply. Straw return simultaneously drove morphological optimization (greater absorption area) and physiological enhancement, leading to stronger bleeding traits and more effective water and nutrient transport. This synergistic effect ultimately supports crop growth and development [29]. Similar results were reported by Kong et al. [12], who discovered that deep tillage with straw return increased subsoil nitrogen content and root distribution, thereby improving nutrient transport to the shoot. PLS-SEM analysis in this study confirmed that straw return primarily regulated bleeding and morphological traits, which indirectly improved canopy physiology and yield formation (Figure 5b). Based on a two-year field study, Che et al. [30]. reported that rice straw return promoted root growth by increasing soil total nitrogen, alkaline-hydrolyzable nitrogen, and available potassium, thereby enhancing soil fertility. In the present study, different straw return methods improved the properties and component levels of root bleeding sap, providing further evidence that straw incorporation promotes root growth and nutrient uptake through improved soil fertility. The path coefficients underscored the central role of root function in this system. The intrinsic mechanism lies in dual improvement, where the increases in root morphological indices (length, surface area, and volume) expanded soil resource capture, while the enhanced bleeding traits reflected greater nitrogen metabolism and long-distance transport capacity [31,32].

### 3.2. From Roots to Canopy: Systemic Responses in Photosynthesis and Carbon–Nitrogen Metabolism

Straw return significantly influences maize photosynthetic traits by modifying soil conditions and nutrient cycling, which regulate root function and canopy carbon-nitrogen metabolism [17,33]. In winter wheat-summer maize systems, different return practices (e.g., incorporation rate, depth, and fragmentation degree) alter soil environments and crop performance [34]. Previous studies have reported that no-till with wheat straw mulching can conserve soil moisture and improve maize photosynthetic physiology [35]. The coordination of carbon assimilation and nitrogen metabolism is complex, and maintaining high activities of carbon- and nitrogen-metabolizing enzymes during silking is essential for delaying senescence, sustaining biomass accumulation, and improving yields [36,37]. In this study, DF significantly increased the chlorophyll content, P_n_, T_r_, and WUE in maize leaves. Simultaneously, all straw return practices enhanced the activities of major carbon- and nitrogen-metabolizing enzymes during silking. These improvements likely resulted from enhanced root bleeding traits and morphological development, which promoted the uptake and transport of water and nutrients. The elevated levels of free amino acids and soluble proteins in the bleeding sap provided abundant substrates for nitrogen metabolism, thereby stimulating enzyme activities such as RuBP and nitrate reductase. The increase in photosynthetic rate reflected not only higher chlorophyll content but also better coordination between nitrogen metabolism and carbon assimilation. The differences in soil structure and decomposition rate among treatments further contributed to the variations in soil water and nutrient availability. These findings were consistent with those of Xiao et al. [38], who reported that deep straw incorporation with plowing improved topsoil nutrient conditions, promoted root development, and consequently enhanced photosynthesis and transpiration in semi-arid Mollisol regions. Similarly, Chen et al. [39] reported that increased tillage depth improved photosynthetic capacity, enzyme activities, and chlorophyll content, jointly contributing to greater carbon assimilation and biomass production. The PLS-SEM model in this study further demonstrated that root bleeding and morphological traits exerted significant effects on enzyme activity, which in turn regulated photosynthetic traits (Figure 5b). These results suggest that root function sustains efficient canopy physiology through a dual mechanism of substrate supply and metabolic signaling [40]. The underlying mechanism is the capacity of a well-developed root system to continuously transport water, nitrogen, and organic compounds to leaves, ensuring sufficient substrates and structural materials for sustained photosynthetic and metabolic activity.

### 3.3. Yield Formation Mechanisms: Statistical and Model Evidence Based on Root–Shoot Interactions

Straw return has been recognized as an effective management strategy for enhancing biomass accumulation and crop productivity. Previous studies have suggested that integrating tillage with straw return in wheat-maize rotation systems significantly improves yield and nutrient use efficiency [41]. Liao et al. [42] further noted that the straw mulching benefited the dry matter accumulation and yield, while its effectiveness depended on mulch thickness, application timing, and planting density. Because most dry matter in maize grains originates from photosynthates produced after silking, post-silking dry matter accumulation is a decisive factor for grain yield [43]. According to Felices Sartori et al. [44], no-till strategies enhance soil physical properties, thereby lowering the threshold for root growth restriction. This finding aligns with our results, in which different straw return methods promoted root growth, providing further evidence that these practices improve the root growth environment. In this study, RF analysis identified root nitrate transport capacity (RNO), bleeding intensity (RBI), root length (RL), root volume (RV), nitrate reductase (NR) activity, and SPAD value as the major yield determinants. This finding indicated that root physiological activity and morphological traits jointly constituted the foundation of yield formation. Partial Least Squares Path Modeling (PLS-SEM) further revealed that straw return directly optimized root bleeding and morphological development, which subsequently enhanced photosynthetic performance. This process significantly promoted post-anthesis nitrogen accumulation and dry matter allocation to grains, thereby increasing yield. The pathway clarified by PLS-SEM underscores the core mechanism behind the yield advantage of DF treatment. Enhanced root function maintained a continuous supply of photosynthetic assimilates after anthesis, ensuring their efficient allocation to grain filling. An increase in kernel weight emerged as the most important contributing factor. These findings are in strong agreement with the concept of “carbon-nitrogen synergistic accumulation driving biomass formation” proposed by Zhang et al. [45] and with the conclusion by Liao et al. [42] that “the effects of straw return are management-dependent”. Moreover, the present results could provide deeper mechanistic insights into yield improvement from the perspective of root-canopy interactions.

## 4. Materials and Methods

### 4.1. Experimental Design

**Figure 6:**
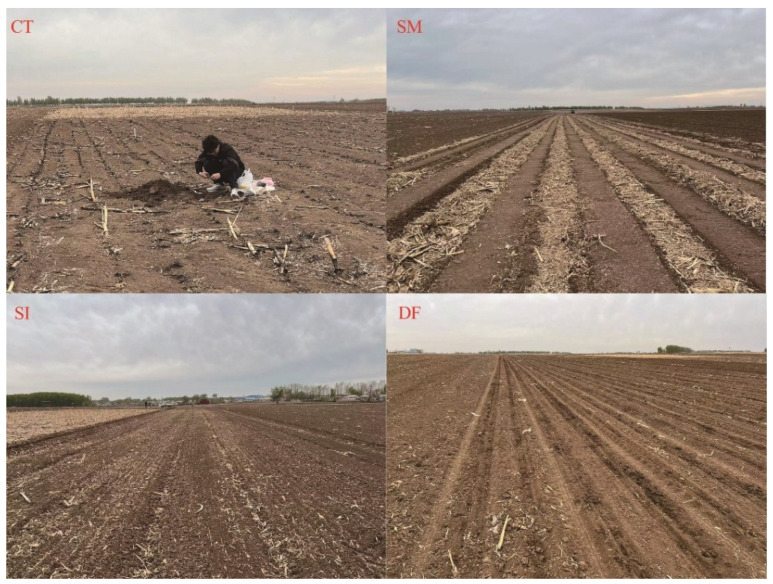
Different treatments before sowing.

A two-year field experiment was conducted from 2023 to 2024 in Dongxing Village (43°31′ N, 124°48′ E), Gongzhuling City, Jilin Province, China. The site has a temperate continental monsoon climate, with a mean annual temperature of 5.6 °C and average precipitation of 594.8 mm. Please refer to Figure S1 in the Supplementary Materials. Prior to the experiment, the physicochemical properties of the topsoil (0–20 cm) prior to the experiment were: pH 7.19, organic matter 20.75 g kg^−1^, available nitrogen 100.92 mg kg^−1^, available phosphorus 14.75 mg kg^−1^, and available potassium 160.67 mg kg^−1^. The soil was classified as Mollisol (Chernozem) with a texture of 22.53% sand, 48.92% silt, and 28.55% clay [46]. The maize (*Zea mays* L.) cultivar ‘Fumin 985’ (128-day growth period) was used. Sowing and harvesting dates were April 25 and September 26 in 2023 and April 28 and September 30 in 2024.The experiment followed a single-factor randomized complete block design (RCBD) with four treatments and three replicates. The treatments were: (1) conventional tillage (CT), where straw was removed and soil was tilled by rotary plowing; (2) straw incorporation (SI), where chopped straw was spread on the soil surface and incorporated into the 0–15 cm layer by rotary tillage; (3) straw mulching (SM), where chopped straw was surface-mulched and a strip tiller was used for row cleaning and seeding; and (4) deep straw incorporation (DF), where chopped straw was buried at 30–35 cm depth with a moldboard plow. For SI, SM, and DF treatments, all straw was returned in situ at full quantity. An on-site schematic is shown in Figure 6. Each experimental plot measured 1200 m^2^. Maize was planted in a wide–narrow row configuration (80 cm wide rows and 40 cm narrow rows). A compound fertilizer (N–P_2_O_5_–K_2_O: 25–10–10) was applied at sowing as a basal dose of 1000 kg ha^−1^, supplying 250 kg N ha^−1^, 100 kg P_2_O_5_ ha^−1^, and 100 kg K_2_O ha^−1^. This rate was designed to ensure that nutrient availability was not a limiting factor for crop growth, thereby allowing a clear assessment of the treatment effects (i.e., different straw return methods) on plant physiology and yield. The planting density was maintained at 60,000 plants ha^−1^. Pest, weed, and disease management was performed uniformly across all plots.

### 4.2. Root Bleeding Sap Collection and Morphological Measurements

**Figure 7:**
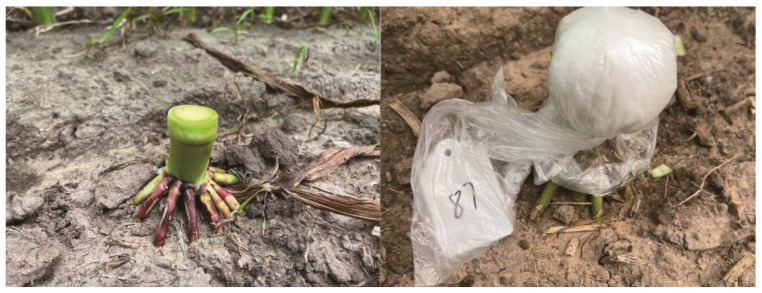
Root Bleeding Sap Collection.

During the silking stage, three representative plants with uniform growth were selected from each plot. The absorbent cotton, plastic bag, and rubber band were weighed before sampling. The stem was cut transversely 3 cm above the root base, and the exposed surface was immediately wrapped with pre-weighed cotton, covered with a plastic bag, and secured with a rubber band. After three hours, the cotton was retrieved and weighed to determine its fresh weight. The on-site schematic is presented in Figure 7. Root bleeding intensity was calculated as: Bleeding Intensity = (Total Weight after Absorption—Total Weight before Absorption)/Absorption Time. The collected bleeding sap was stored at −80 °C for subsequent biochemical analysis.

Ammonium nitrogen content was determined using the indophenol blue method [47], nitrate nitrogen by ultraviolet spectrophotometry [48], soluble protein by the Coomassie Brilliant Blue G-250 method [49], soluble sugar by the phenol–sulfuric acid method [50], and free amino acids by the ninhydrin coloration method [51].

During silking, root samples were collected from three random locations in each plot. The roots were carefully excavated, rinsed with clean water to remove soil, and scanned using a flatbed scanner (Expression 11000XL, Epson, China). Morphological parameters, including total root length, surface area, mean diameter, volume, and tip number, were analyzed using WinRHIZO Pro 32-bit 2013c (Regent Instruments Inc., Québec City, QC, Canada). Roots were then oven-dried at 80 °C to a constant weight to determine the root-shoot ratio, which was calculated as belowground dry weight divided by aboveground dry weight.

### 4.3. Leaf Photosynthetic Measurements

The relative chlorophyll content (SPAD value) was measured at the jointing, bell, silking, and milk stages using a portable chlorophyll meter (SPAD-502, Konica Minolta, Tokyo, Japan). During silking, the net photosynthetic rate (P_n_), stomatal conductance (G_s_), intercellular CO_2_ concentration (C_i_), and transpiration rate (T_r_) were determined for the ear leaves of five representative plants per plot. Measurements were performed on clear mornings between 9:00 and 11:00 under steady-state light conditions. An open-path infrared gas analyzer (LI-6400XT, LI-COR Biosciences, Bourne, MA, USA) was used. WUE was calculated as the ratio of P_n_ to T_r_.

### 4.4. Leaf carbon and nitrogen metabolism enzymes

At the silking stage, a fresh portion of ear leaves was collected from each plot. The samples were immediately flash-frozen in liquid nitrogen and transported to the laboratory, where they were stored at −80 °C until enzyme activity analysis. The activities of RuBP, PEP, nitrate reductase (NR), and glutamine synthetase (GS) were quantified using commercial assay kits (Suzhou Michy Biomedical Technology Co., Ltd., Jiangsu, China) with an enzyme microplate reader (SpectraMax i3x, Molecular Devices, LLC, Urstein, Austria). The assay procedures followed the manufacturer’s instructions, which were obtained from the company’s website (www.michybio.com) and included brief descriptions of the assay principles and enzyme activity unit definitions.

### 4.5. Nitrogen Accumulation and Translocation

At the silking and milk stages, three plants per plot were separated into stems, leaves, sheaths, and ears (silking) or stems, leaves, sheaths, bracts, cobs, and grains (maturity). All tissues were oven-dried, and nitrogen concentration was determined using the Kjeldahl method [52]. Nitrogen accumulation (kg·ha^−1^) = dry weight per plant × planting density × nitrogen content per plant. Vegetative organ nitrogen translocation (kg·ha^−1^) = nitrogen accumulation at silking—nitrogen accumulation at maturity. Translocation rate (%) = (translocation amount/silking accumulation) × 100.

### 4.6. Dry Matter Accumulation

At the jointing, bell, silking, milk, and maturity stages, three plants from each treatment were sampled, oven-dried at 105 °C for 30 min, and maintained at 80 °C until a constant weight was achieved. The dried samples were weighed to determine dry matter accumulation.

### 4.7. Yield and Yield Components

At maturity, ears were collected from three 10 m^2^ areas per plot. The yield components, including kernel number per ear, 100-kernel weight, and seed-setting rate, were determined using ten randomly selected ears. Grain yield was adjusted to a standard moisture content of 14%.

### 4.8. Statistical Analysis

All data were expressed as the mean ± standard error. Preliminary data statistics and organization were performed using Excel 2021, and all statistical analyses were performed using SPSS 27.0 (IBM, Armonk, NY, USA). Treatment effects were evaluated using one-way analysis of variance (ANOVA), and mean comparisons among treatments were conducted using Duncan’s multiple range test (DMRT) and the LSD test, with a significance level of *p* < 0.05. Year × treatment interactions were assessed through two-way ANOVA. Random forest (RF) modeling was implemented in R 4.4.2 with the ‘rfPermute’ and ‘rfUtilities’ packages (ntree = 5000, seed = 500) to identify yield drivers. Causal relationships among latent variables were examined using partial least squares path modeling (PLS-SEM) via the ‘plspm’ package in R, retaining only paths with *p* < 0.05. All figures were prepared using Origin 2021 (OriginLab, Northampton, MA, USA).

## 5. Conclusions

Different straw return practices markedly influenced maize root bleeding traits, morphology, photosynthesis, carbon-nitrogen metabolism, biomass accumulation, and yield. Among them, deep straw incorporation (DF) most effectively enhanced root physiological activity (bleeding intensity and solute concentration in bleeding sap) and root morphology (length, surface area, and volume), thereby improving absorptive capacity. DF also increased leaf chlorophyll content, photosynthetic rate, water use efficiency, and carbon-nitrogen enzyme activities, ensuring a sufficient photoassimilate supply. Consequently, DF promoted dry matter and nitrogen accumulation, particularly during silking and milk stages, facilitated translocation to grains, and significantly improved yield by increasing kernel weight. PLS-SEM analysis confirmed that root bleeding and morphological traits synergistically regulated photosynthetic metabolism and drove yield formation via biomass accumulation. These findings emphasize the importance of straw return practices in enhancing root function and crop productivity, thereby supporting sustainable tillage in the Songnen Plain. Future research should extend the observation period and integrate soil microbiological and plant physiological approaches to further clarify straw-soil-crop feedback mechanisms and optimize straw return management.

## Figures and Tables

**Table 1:** Enzyme activities of leaf carbon and nitrogen metabolism.

Year	Treatment	RuBP Carboxylase (U·g^−1^)	PEP Carboxylase (U·g^−1^)	NR Activity (µg·g^−1^·h^−1^)	GS Activity (A·mg^−1^·h^−1^)
2023	CT	59.11 ± 2.39 c	6.26 ± 0.16 d	28.54 ± 0.64 c	11.17 ± 0.36 c
	SI	61.22 ± 2.72 bc	7.40 ± 0.24 b	35.17 ± 0.88 b	11.47 ± 0.30 bc
	SM	64.44 ± 2.09 b	6.96 ± 0.15 c	33.21 ± 1.27 b	11.94 ± 0.34 b
	DF	69.51 ± 2.72 a	8.35 ± 0.22 a	38.39 ± 1.13 a	12.19 ± 0.47 a
2024	CT	50.62 ± 3.23 b	7.07 ± 0.23 c	29.40 ± 0.40 c	9.98 ± 0.12 c
	SI	56.78 ± 1.36 b	7.82 ± 0.15 b	33.77 ± 0.85 b	10.54 ± 0.18 b
	SM	68.37 ± 5.26 a	7.77 ± 0.18 ab	33.77 ± 0.85 b	10.84 ± 0.31 b
	DF	64.56 ± 3.57 a	8.17 ± 0.21 a	36.86 ± 0.76 a	11.56 ± 0.27 a
Year (Y)		*	**	NS	**
Treatment (T)		**	**	**	**
(Y) × (T)		NS	*	NS	NS

* and ** indicate significance at the 0.05 and 0.01 levels, respectively. NS denotes non-significant. Identical letters within the same column indicate no significant difference at the 0.05 level. Different lowercase letters indicate significant differences (*p* < 0.05).

**Table 2:** Nitrogen Accumulation and Translocation in Maize Vegetative Organs.

Year	Treatment	Accumulation Before Silking (kg/hm^2^)	Accumulation After Silking (kg/hm^2^)	Volume of Transshipment (kg/hm^2^)	Transport Rate (%)
2023	CT	127.99 ± 2.35 c	70.34 ± 1.89 c	57.65 ± 4.40 a	44.98 ± 1.92 a
	SI	133.66 ± 1.93 b	75.30 ± 2.37 bc	58.35 ± 4.20 a	43.62 ± 2.55 a
	SM	132.34 ± 2.50 b	76.87 ± 3.89 b	55.46 ± 2.45 a	41.93 ± 2.19 a
	DF	145.18 ± 2.93 a	85.61 ± 1.38 a	59.57 ± 2.84 a	41.00 ± 1.12 a
2024	CT	107.88 ± 3.38 b	48.15 ± 3.38 c	59.73 ± 1.27 a	54.31 ± 2.24 a
	SI	111.51 ± 1.61 b	56.06 ± 3.38 b	55.45 ± 4.96 a	49.67 ± 3.73 a
	SM	112.78 ± 2.08 b	55.78 ± 1.28 b	57.00 ± 5.54 a	50.49 ± 3.32 a
	DF	126.30 ± 3.17 a	64.89 ± 2.81 a	61.40 ± 7.31 a	48.47 ± 3.90 a
Year (Y)		**	**	NS	**
Treatment (T)		**	**	NS	NS
(Y) × (T)		NS	NS	NS	NS

* and ** indicate significance at the 0.05 and 0.01 levels, respectively. NS denotes non-significant. Identical letters within the same column indicate no significant difference at the 0.05 level. Different lowercase letters indicate significant differences (*p* < 0.05).

**Table 3:** Yields and Yield Components.

Year	Treatment	Number of Grains	Seed Yield (%)	100-Grain Weight (g)	Yield (kg/hm^2^)
2023	CT	578.40 ± 85.63 a	88.14 ± 0.40 a	35.38 ± 0.20 c	11195.80 ± 144.96 b
	SI	590.26 ± 61.18 a	88.22 ± 0.32 a	37.53 ± 0.24 b	11283.24 ± 207.27 b
	SM	599.06 ± 77.99 a	87.85 ± 0.65 a	38.31 ± 0.43 ab	11469.29 ± 136.16 ab
	DF	608.00 ± 49.18 a	88.23 ± 0.13 a	38.79 ± 0.60 a	11737.36 ± 180.59 a
2024	CT	515.73 ± 65.78 a	87.44 ± 1.53 a	33.07 ± 0.10 c	11099.46 ± 229.48 c
	SI	528.00 ± 79.71 a	86.61 ± 0.75 a	34.67 ± 0.32 b	11164.80 ± 300.94 bc
	SM	536.53 ± 44.41 a	87.69 ± 0.66 a	34.54 ± 0.65 b	11790.14 ± 297.92 ab
	DF	556.80 ± 76.58 a	88.50 ± 0.35 a	37.80 ± 0.17 a	12415.01 ± 302.27 a
Year (Y)		NS	NS	**	NS
Treatment (T)		NS	NS	**	**
(Y) × (T)		NS	NS	**	*

* and ** indicate significance at the 0.05 and 0.01 levels, respectively. NS denotes non-significant. Identical letters within the same column indicate no significant difference at the 0.05 level. Different lowercase letters indicate significant differences (*p* < 0.05).

## Data Availability

Data are contained within the article.

## Supplementary Materials

The following supporting information can be downloaded at: https://www.mdpi.com/article/10.3390/plants14213331/s1, Figure S1. Description of the Experimental Site and Climate Conditions. Figure S2. Field Schematic Diagrams of Different Straw Returning Methods Before Sowing. Figure S3. Root Bleeding Sap Collection. Figure S4. Aerial view of the test site. Figure S5. Soil profile photographs.

## References

[1] Erenstein O., Jaleta M., Sonder K., Mottaleb K., Prasanna B.M. (2022). Global maize production, consumption and trade: Trends and R&D implications. Food Secur..

[2] Yan Z., Zhiyuan T., Rui M., Lili Q., Yihang W. (2025). Impact of soil erosion-driven degradation on crop yield in black soils of Northeast China’s Songnen Plain. Pedosphere.

[3] Wang H., Shen M., Hui D., Chen J., Sun G., Wang X., Lu C., Sheng J., Chen L., Luo Y. (2019). Straw incorporation influences soil organic carbon sequestration, greenhouse gas emission, and crop yields in a Chinese rice (*Oryza sativa* L.)–wheat (*Triticum aestivum* L.) cropping system. Soil Tillage Res..

[4] Peng Z., Wang L., Xie J., Li L., Coulter J.A., Zhang R., Luo Z., Cai L., Carberry P., Whitbread A. (2020). Conservation tillage increases yield and precipitation use efficiency of wheat on the semi-arid Loess Plateau of China. Agric. Water Manag..

[5] Wei X., He K., Ma B.-L., Guo S., Feng C., Liu C., Ma Y., Li P. (2025). Straw return significantly enhances wheat yield in higher precipitation environment by promoting larger root diameters, wider xylem channels, and thinner root cortex. Plant Soil.

[6] Wang X.-X., Li J., Wang D., An T., Qin W., Zou H., Rengel Z. (2022). Straw incorporation effects on net photosynthetic carbon assimilation and maize growth. Front. Agron..

[7] Zhao R., Li Y., Chen X., Zhang Z., Zhou Z., Zhou Y., Qi Z. (2024). Heterosis Analysis in Endogenous Substances in Root Bleeding Sap of Sorghum. Phyton.

[8] Wang J., Fan J., Wang H., Wang X., Xing Y., Gao Y., Hao M. (2025). Dual-mulching under no-tillage promotes maize root growth and improves yield by optimizing soil hydrothermal conditions in semi-arid regions. Agric. Water Manag..

[9] Wang Y., Yu A., Wang P., Shang Y., Wang F., Lyu H., Pang X., Li Y., Liu Y., Yin B. (2025). No-tillage with total green manure mulching can improve soil moisture and temperature environment, promote maize root structure and photosynthetic capacity to increase maize yield. J. Integr. Agric..

[10] Chen X., Ren H., Zhang J., Zhao B., Ren B., Wan Y., Liu P. (2024). Deep phosphorus fertilizer placement increases maize productivity by improving root-shoot coordination and photosynthetic performance. Soil Tillage Res..

[11] Diacono M., Baldivieso-Freitas P., Sans Serra F.X. (2019). Nitrogen utilization in a cereal-legume rotation managed with sustainable agricultural practices. Agron.J..

[12] Kong F., Jiu A., Kan Z., Zhou J., Yang H., Li F.-M. (2024). Deep tillage combined with straw biochar return increases rice yield by improving nitrogen availability and root distribution in the subsoil. Field Crops Res..

[13] Dong S., Bismark A.-B., Li S., Gao Q., Zhou X., Li C. (2024). Ammonium Polyphosphate Promotes Maize Growth and Phosphorus Uptake by Altering Root Properties. Plants.

[14] Li Y., Wang Q., Gao S., Wang X., He A., He P. (2025). Effects of water–nitrogen coupling on root distribution and yield of summer maize at different growth stages. Plants.

[15] Wang Z., Sun J., Du Y., Niu W. (2022). Conservation tillage improves the yield of summer maize by regulating soil water, photosynthesis and inferior kernel grain filling on the semiarid Loess Plateau, China. J. Sci. Food Agric..

[16] Buivydienė A., Deveikytė I., Veršulienė A., Feiza V. (2024). Tillage Practices Effect on Root Distribution and Variation of Soil CO_2_ Emission under Different Cropping Strategies. Agron. J..

[17] Wu G., Ling J., Liu Z.-X., Xu Y.-P., Chen X.-M., Wen Y., Zhou S.-L. (2022). Soil warming and straw return impacts on winter wheat phenology, photosynthesis, root growth, and grain yield in the North China Plain. Field Crops Res..

[18] Li X., Wang H., Sun S., Ji X., Wang X., Wang Z., Shang J., Jiang Y., Gong X., Qi H. (2024). Optimization of the morphological, structural, and physicochemical properties of maize starch using straw returning and nitrogen fertilization in Northeast China. Int. J. Biol. Macromol..

[19] Jin W., Liu Z., Cheng Z., Wang Q., Hu W., Chen B., Meng Y., Zhou Z. (2024). The trade-off between root growth redundancy and premature senescence under different straw returning modes affects boll formation and seedcotton yield. Eur. J. Agron..

[20] Liu R.-Z., Borjigin Q., Gao J.L., Yu X.F., Hu S.P., Li R.-P. (2024). Effects of different straw return methods on soil properties and yield potential of maize. Sci. Rep..

[21] Lian Y., Zhang X., Du F., Zhang X., Ali S. (2024). Mulch drip fertigation with diverse tillage practices regulating root bleeding sap, root growth, lodging resistance and improve maize productivity. Agric. Water Manag..

[22] Jia Q., Chen K., Chen Y., Ali S., Sohail A., Fahad S. (2018). Mulch covered ridges affect grain yield of maize through regulating root growth and root-bleeding sap under simulated rainfall conditions. Soil Tillage Res..

[23] Ma L., Kong F., Wang Z., Luo Y., Lv X., Zhou Z., Meng Y. (2019). Growth and yield of cotton as affected by different straw returning modes with an equivalent carbon input. Field Crops Res..

[24] Zhang W., Long A., Ji X., Sun Z., Tian P., Jin C., Gong X., Jiang Y., Qi H., Yu H. (2026). Tillage combined with straw return increases maize yield and water use by regulating root morphological distribution and nitrogen metabolism in Northeast China. Soil Tillage Res..

[25] Guo S., Liu Z., Zhou Z., Lu T., Chen S., He M., Zeng X., Chen K., Yu H., Shangguan Y. (2022). Root system architecture differences of maize cultivars affect yield and nitrogen accumulation in Southwest China. Agriculture.

[26] Liu X., Zhang L., Yu Y., Qian C., Li C., Wei S., Li C., Gu W. (2022). Nitrogen and chemical control management improve yield and quality in high-density planting of maize by promoting root-bleeding sap and nutrient absorption. Front. Plant Sci..

[27] Sun Y., Xie J., Hou H., Li M., Wang Y., Wang X. (2023). Effects of zeolite on physiological characteristics and grain quality in rice under alternate wetting and drying irrigation. Water.

[28] Wen Z., Li H., Shen J., Rengel Z. (2017). Maize responds to low shoot P concentration by altering root morphology rather than increasing root exudation. Plant Soil.

[29] Wang H., Xu R., Li Y., Yang L., Shi W., Liu Y., Chang S., Hou F., Jia Q. (2019). Enhance root-bleeding sap flow and root lodging resistance of maize under a combination of nitrogen strategies and farming practices. Agric. Water Manag..

[30] Che W., Piao J., Gao Q., Li X., Li X., Jin F. (2023). Response of soil physicochemical properties, soil nutrients, enzyme activity and rice yield to rice straw returning in highly saline-alkali paddy soils. Soil Sci. Plant Nutr..

[31] Chen X., Zhu Y., Ding Y., Pan R., Shen W., Yu X., Xiong F. (2021). The relationship between characteristics of root morphology and grain filling in wheat under drought stress. PeerJ.

[32] Xu G.w., Song K.j., Lu D.K., Wang H.Z., Chen M.c. (2019). Influence of water management and nitrogen application on rice root and shoot traits. Agron. J..

[33] Wang J., Hussain S., Sun X., Zhang P., Javed T., Dessoky E.S., Ren X., Chen X. (2022). Effects of nitrogen application rate under straw incorporation on photosynthesis, productivity and nitrogen use efficiency in winter wheat. Front. Plant Sci..

[34] Cui H., Luo Y., Chen J., Jin M., Li Y., Wang Z. (2022). Straw return strategies to improve soil properties and crop productivity in a winter wheat-summer maize cropping system. Eur. J. Agron..

[35] Guo Y., Fan H., Li P., Wei J., Qiu H. (2023). Photosynthetic physiological basis of no tillage with wheat straw returning to improve maize yield with plastic film mulching in arid irrigated areas. Plants.

[36] Reguera M., Peleg Z., Abdel-Tawab Y.M., Tumimbang E.B., Delatorre C.A., Blumwald E. (2013). Stress-induced cytokinin synthesis increases drought tolerance through the coordinated regulation of carbon and nitrogen assimilation in rice. Plant Physiol..

[37] Li R., Hu D., Ren H., Yang Q., Dong S., Zhang J., Zhao B., Liu P. (2022). How delaying post-silking senescence in lower leaves of maize plants increases carbon and nitrogen accumulation and grain yield. Crop J..

[38] Xiao Y., Luo W., Yang K., Fu J., Wang P. (2025). Plow tillage with buried straw increases maize yield by regulating soil properties, root growth, photosynthetic capacity, and bacterial community assembly in semi-arid black soil farmlands. Eur. J. Agron..

[39] Chen H., Zhang X., Zhang S., Liu Z., Liu Z., Shao X., Guo L., Geng Y., Wang L., Lv Y. (2025). Mechanisms of topsoil depth drive differences in maize yield and photosynthetic carbon assimilation. Integr. Agric..

[40] Pan S., Liu H., Mo Z., Patterson B., Duan M., Tian H., Hu S., Tang X. (2016). Effects of nitrogen and shading on root morphologies, nutrient accumulation, and photosynthetic parameters in different rice genotypes. Sci. Rep..

[41] Huang M., Xiao H., Zhang J., Li S., Peng Y., Guo J.-H., Jiang P., Wang R., Chen Y., Li C. (2025). Effects of Long-Term Positioning Tillage Method and Straw Management on Crop Yield and Nutrient Accumulation and Utilization in Dryland Wheat–Maize Double-Cropping System. Agron. J..

[42] Liao C., Tang M., Zhang C., Deng M., Li Y., Feng S. (2025). Impacts of Various Straw Mulching Strategies on Soil Water, Nutrients, Thermal Regimes, and Yield in Wheat–Soybean Rotation Systems. Plants.

[43] Fan P., Ming B., Evers J.B., Li Y., Li S., Xie R., Anten N.P. (2023). Nitrogen availability determines the vertical patterns of accumulation, partitioning, and reallocation of dry matter and nitrogen in maize. Field Crops Res..

[44] Sartori F., Piccoli I., Polese R., Berti A. (2022). Transition to conservation agriculture: How tillage intensity and covering affect soil physical parameters. Soil.

[45] Zhang Y., Wang J., Gong S., Xu D., Mo Y. (2019). Straw mulching enhanced the photosynthetic capacity of field maize by increasing the leaf N use efficiency. Agric. Water Manag..

[46] USDA (1954). Diagnosis and Improvement of Saline and Alkali Soils.

[47] Solorzano L. (1969). Determination of Ammonia in Natural Waters by the Phenolhypochlorite Method. Master’s Thesis.

[48] Cawse P. (1967). The determination of nitrate in soil solutions by ultraviolet spectrophotometry. Analyst.

[49] Kochert G. (1978). Carbohydrate determination by the phenol-sulfuric acid method. Handbook of Phycological Methods, Physiological and Biochemical Methods.

[50] Bradford M.M. (1976). A rapid and sensitive method for the quantitation of microgram quantities of protein utilizing the principle of protein-dye binding. Anal. Biochem..

[51] Bates L.S., Waldren R., Teare I. (1973). Rapid determination of free proline for water-stress studies. Plant Soil.

[52] Dai H.L., Wu X.J. (1995). The nitrogen content determined in dry plant samples by Kjeldahl method. Jiangsu Agric. Res..

