# Supplementary material for: Physiological Mechanisms Underlying Maize Yield Enhancement by Straw Return in the Thin-Layer Mollisol Region of the Songnen Plain
Source: Plants (Basel). 2025 Oct 31;14(21):3331. doi: 10.3390/plants14213331 (PMC12609241; doi:10.3390/plants14213331)
Supplement: Supplementary file 1 [file plants-14-03331-s001.zip › plants-3922882-supplementary.pdf]

Dear Editors and Experts,

I will briefly introduce our experimental field and the specific farming methods employed.

A two-year field experiment was conducted from 2023 to 2024 in Dongxing Village (43°31′N, 124°48′E), Gongzhuling City, Jilin Province, China. The site has a temperate continental monsoon climate, with a mean annual temperature of 5.6°C and average precipitation of 594.8 mm.

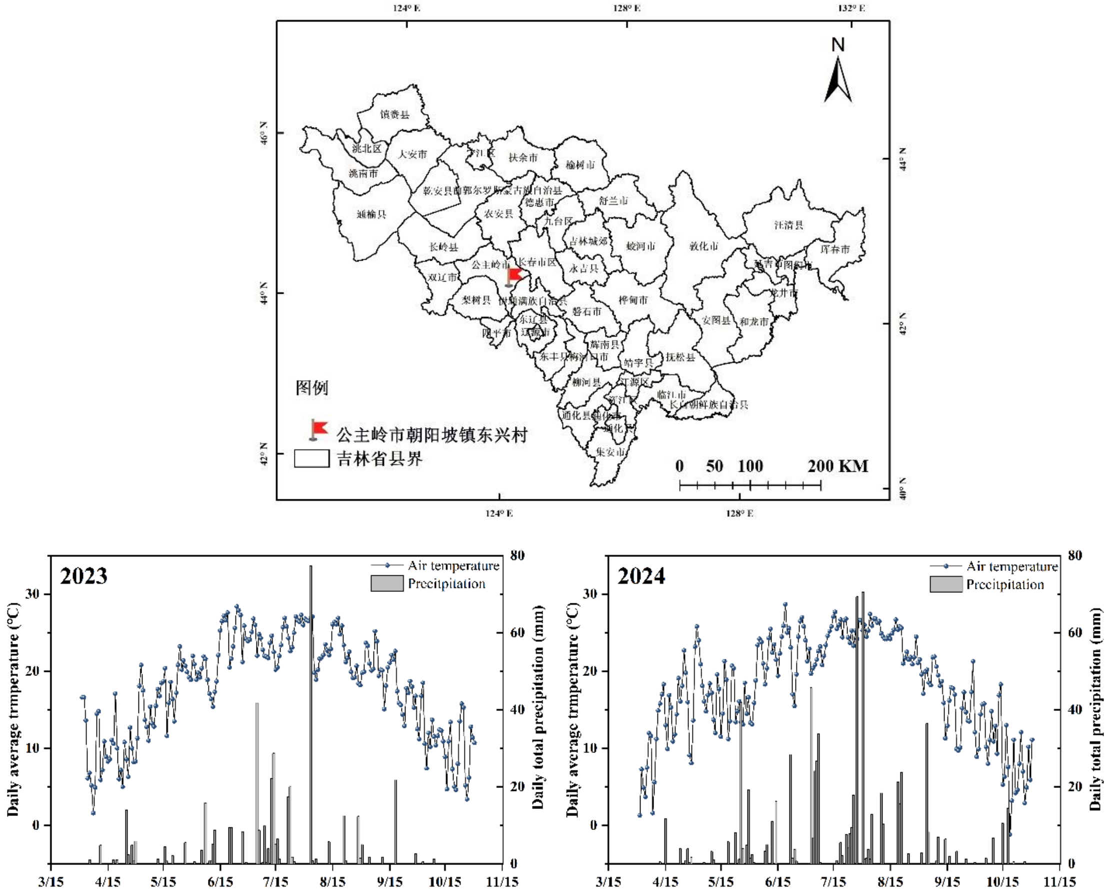

Figure S1. Description of the Experimental Site and Climate Conditions

Below are images of the tillage sites for each treatment. The treatments were (1) conventional tillage (CT), where straw was removed and soil was tilled by rotary plowing; (2) straw incorporation (SI), where chopped straw was spread on the soil surface and incorporated into the 0–15 cm layer by rotary tillage; (3) straw mulching (SM), where chopped straw was surface-mulched and a strip tiller was used for row cleaning and seeding; and (4) deep straw incorporation (DF), where chopped straw was buried at 30–35 cm depths with a moldboard plow. For SI, SM, and DF treatments, all straw was returned in situ at full quantity.

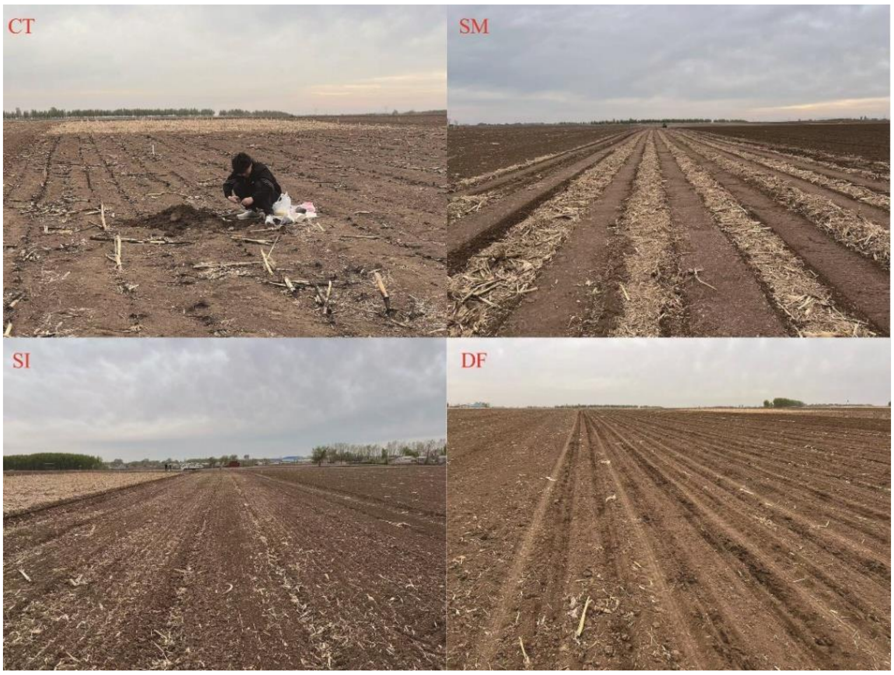

Figure S2. Field Schematic Diagrams of Different Straw Returning Methods Before Sowing

During the silking stage, three representative plants with uniform growth were selected from each plot. The absorbent cotton, plastic bag, and rubber band were weighed before sampling. The stem was cut transversely 3 cm above the root base, and the exposed surface was immediately wrapped with pre-weighed cotton, covered with a plastic bag, and secured with a rubber band. After three hours, the cotton was retrieved and weighed to determine its fresh weight.

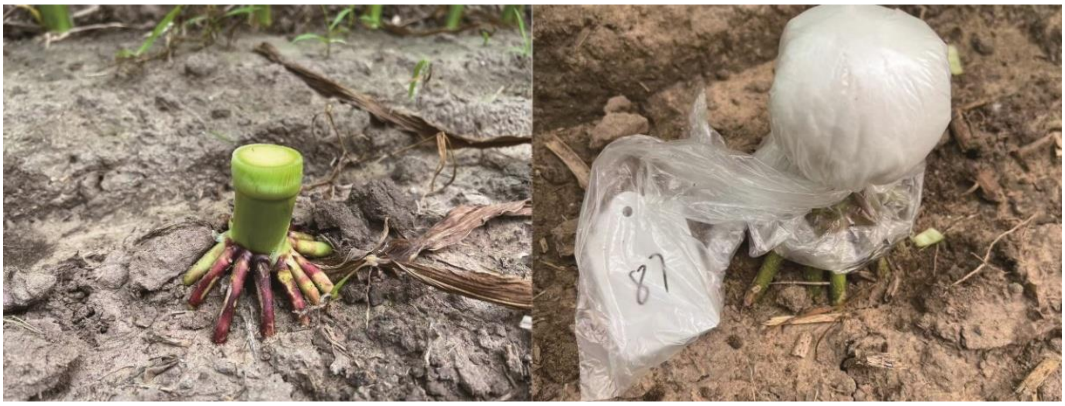

Figure S3. Root Bleeding Sap Collection

The results could establish a theoretical foundation for efficient straw return technologies to improve maize yield and resource-use efficiency in the thin-layer Mollisol region of Northeast China.

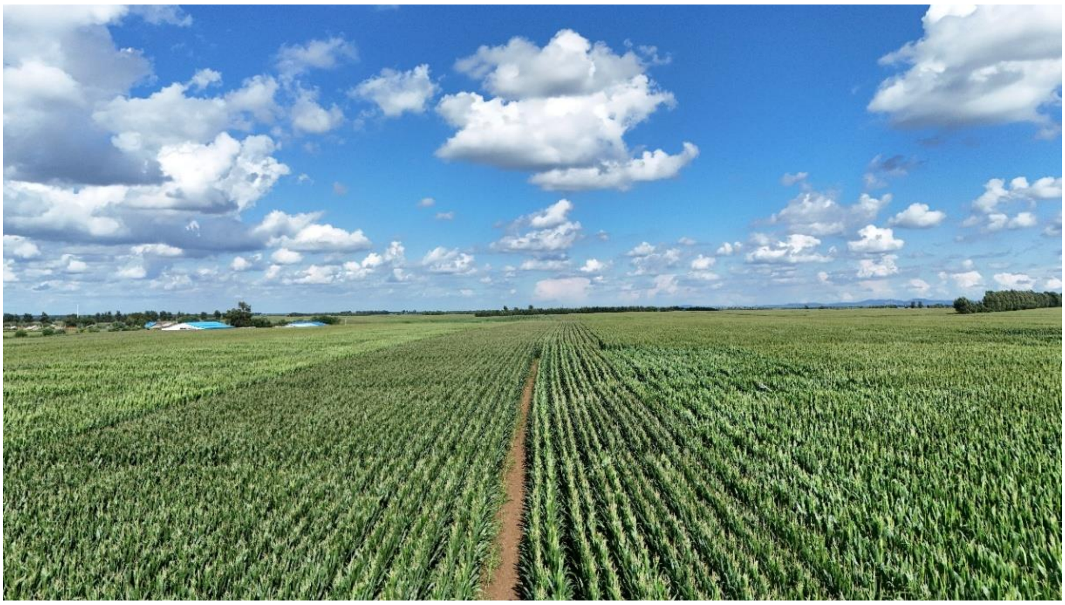

Figure S4. Aerial view of the test site

The parent material at our experimental site in Chaoyangpo Town, Gongzhuling City, Jilin Province, is alluvial deposits. The soil is classified as Clayey Gravelly Black Soil, a subcategory of the Black Soil series, with a profile sequence of A11-Ah-AhC-C This representative soil profile photograph was taken during the initial site characterization on October 9, 2022. The photo has been added to the attachment.

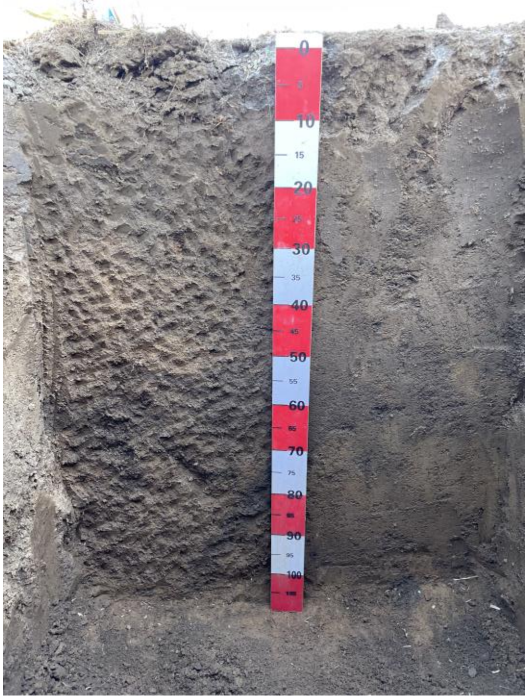

Figure S5. Soil profile photographs
